# Association of Polymorphisms in Inflammation Genes With the Prognosis of Advanced Non-Small Cell Lung Cancer Patients Receiving Epidermal Growth Factor Receptor Tyrosine Kinase Inhibitors

**DOI:** 10.3389/fonc.2022.836117

**Published:** 2022-03-18

**Authors:** Xuelin Zhang, Tengfei Ye, Mingdong Li, Hongwang Yan, Hui Lin, Hongsheng Lu, Zecheng Qi, Haihui Sheng, Chunya He

**Affiliations:** ^1^Department of Thoracic Surgery, The First People’s Hospital of Wenling, Taizhou, China; ^2^Department of Pharmacy, The First People’s Hospital of Wenling, Taizhou, China; ^3^Department of Pathology, Taizhou Central Hospital, Taizhou, China; ^4^Department of Thoracic Surgery, Taizhou Central Hospital, Taizhou, China; ^5^Outdo Clinic, Shanghai Engineering Center for Molecular Medicine, National Engineering Center for Biochip at Shanghai, Shanghai, China; ^6^Department of Surgical Oncology, Taizhou Central Hospital, Taizhou, China

**Keywords:** lung cancer, epidermal growth factor receptor, tyrosine kinase inhibitor, inflammation, single nucleotide polymorphism

## Abstract

**Background:**

Inflammation is not only involved in the development and progression of cancer but also affects the response to therapy. The aim of this study was to investigate the association of single nucleotide polymorphisms (SNPs) in inflammation genes with the prognosis of advanced non-small cell lung cancer (NSCLC) patients treated with epidermal growth factor receptor (EGFR) tyrosine kinase inhibitors (TKIs).

**Methods:**

Forty-seven SNPs were genotyped in 318 advanced NSCLC patients receiving EGFR-TKIs. Of 318 patients, 182 (57.2%) patients died during follow-up period. We assessed the association of SNPs with the progression-free survival (PFS) and overall survival (OS) as well as calculated the weighted genetic risk score (GRS). We also explored the expression levels and prognostic values of inflammation genes in lung adenocarcinoma (LUAD) in Gene Expression Profiling Interactive Analysis (GEPIA) and using UCSC Xena, respectively. The relationship between the expression levels of IL15, IL17RA, AGER, MIF, and TNFRSF1A and EGFR mutation status was analyzed using UCSC Xena.

**Results:**

In single variant analyses, 3 SNPs (rs10519613, rs4819554, and rs4149570) were significantly associated with worse PFS. Five SNPs (rs10519613, rs4819554, rs2070600, rs755622, and rs4149570) were significantly with worse OS. In addition, high and intermediate GRSs (based on rs10519613, rs4819554, and rs4149570) were associated with worse PFS than those with low GRS. For OS, patients with high GRSs (based on rs10519613, rs4819554, rs2070600, rs755622, and rs4149570) had shorter survival time than those with low GRS. Furthermore, IL15, IL17RA, AGER, MIF, and TNFRSF1A were dysregulated in LUAD. There was difference in the expression level of TNFRSF1A between EGFR wildtype and EGFR-mutant LUAD. Both low AGER expression and high TNFRSF1A expression were significantly associated with worse PFS in LUAD. In addition, low IL17RA and AGER expression, high MIF and TNFRSF1A expression were significantly associated with worse OS in LUAD.

**Conclusion:**

SNPs in inflammation genes could serve as prognostic biomarkers for NSCLC patients treated with EGFR-TKIs.

## Introduction

Lung cancer is the second most common cancer and the leading cause of cancer death worldwide, with 2.2 million new cases and 1.8 million deaths annually, accounting for almost 1 in 10 (11.4%) cases and 1 in 5 (18.0%) cancer deaths globally (1). China is a country with a high incidence of lung cancer, ranking the first for both incidence and mortality (2). The incidence and mortality rates of lung cancer remain high for many years. Whether high incidence or high mortality rates of lung cancer undoubtedly make it a major problem of public health in China. At present, most of metastatic lung cancer still cannot be cured. Although the combined application of surgery, chemotherapy, radiotherapy, and targeted therapy has significantly improved the survival rate of lung cancer patients, the prognosis of lung cancer is still poor (3). For example, the 5-year overall survival rate of TNM stage IA lung cancer is about 85%, while that of TNM stage IV lung cancer is only 6% (4, 5). The most effective treatment strategy could be selected by analyzing molecular markers to guide individualized treatment, which can maximize the efficacy and minimize toxicity, and achieve the goal of improving the survival rate and quality of life of patients.

Epidermal growth factor receptor (EGFR) mutations are present in about ~50.2% of non-small cell lung cancer (NSCLC) (6, 7). Tyrosine kinase inhibitors (TKIs) targeting EGFR have made great progress in the treatment of NSCLC patients harboring activating EGFR mutations. TKIs have obviously increased the disease-specific survival for NSCLC patients (8, 9). To date, first-, second-, and third-generation EGFR-TKIs are approved as the standard first-line treatments for EGFR-mutated patients (10, 11). However, the objective response rate of EGFR-TKIs is approximately ~80%, indicating that at least 20% of patients may not benefit from EGFR-TKI therapy (12, 13). Acquired resistance inevitably develops in most patients receiving EGFR-TKIs within 1 to 2 years of therapy (12, 14). Patients who failed EGFR-TKIs have little choice other than cytotoxic chemotherapy due to very limited kinds of targeted drugs. Therefore, it is necessary to develop therapeutic strategies or discover drugs that delay the emergence of EGFR-TKI resistance or reverse this effect and to identify more novel predictive biomarkers that can accurately predict the efficacy of EGFR-TKIs.

Inflammation is not only involved in the occurrence, progression, angiogenesis, invasion and metastasis of cancer (15, 16), but is also closely related to 15% of cancer deaths (17). The lung is the most common site of inflammation due to environmental exposure. Pulmonary inflammatory diseases such as chronic obstructive pulmonary disease are associated with increased risk and poor prognosis of lung cancer (18, 19). Many studies have shown that inflammatory molecules and their effectors are independent risk factors for disease progression and survival of advanced lung cancer (20–24). Circulating inflammatory markers such as C-reactive protein (CRP), cytokines, neutrophil-to-lymphocyte ratio (NLR), and lymphocyte-to-monocyte ratio (LMR) are associated with the prognosis of NSCLC patients receiving EGFR-TKIs (20, 23, 25–28). A recent study by Yamaoka et al. (29) found that high level of tumor necrosis factor (TNF) may enhance the development of EGFR TKI-induced pneumonitis, which was responsible for more than half of the toxic deaths following the administration of EGFR-TKIs (30). The clinical outcomes of NSCLC patients receiving EGFR-TKIs appear to be influenced by the degree of systemic inflammation at diagnosis. Single nucleotide polymorphisms (SNPs) may affect the expression and function of inflammation genes. Therefore, SNPs in inflammation genes may be associated with clinical outcome of NSCLC patients treated with EGFR-TKIs. The aim of this study was to investigate the effect of SNPs inflammation genes on the prognosis of patients with advanced NSCLC patients treated with EGFR TKIs.

## Materials and Methods

### Patients

A total of 318 advanced NSCLC patients with EGFR mutations were recruited at Taizhou Central Hospital between 2015 and 2017. All patients must meet the following inclusion criteria: 1) histologically or cytologically confirmed TNM stages IIIB and IV NSCLC; 2) aged 20 and older; 3) activating EGFR mutations; 4) Eastern Cooperative Oncology Group (ECOG) performance status (PS) of 0, 1 or 2; 5) no history of systemic anticancer therapy including chemotherapy and radiotherapy. Patients were initially treated with first-generation EGFR-TKIs orally daily until either intolerable toxicity or disease progression occurred. In addition, if patients developed severe adverse effects, EGFR-TKIs doses were appropriately reduced. The study was approved by the Ethics Committees of Taizhou Central Hospital and all patients provided signed informed consent.

### Genotyping

Fourth-seven inflammation genes were selected based on a literature review. For each candidate gene, SNPs associated with human disease susceptibility, prognosis, and treatment outcomes were searched in PubMed. For the coding SNPs of candidate genes not reported in the literature, those with allele frequency greater than 0.05 in Chinese population were selected. Furthermore, only SNPs with a minimum allele frequency (MAF) greater than 0.05 were selected for genotyping in order to improve the statistical efficiency. Finally, a total of 47 SNPs were selected from 47 inflammation genes (**Table S1**). Genomic DNA was extracted from whole blood using the TaKaRa MiniBEST Whole Blood Genomic DNA Extraction Kit (TakaRa, Dalian, China) according to the manufacturer’s instructions. All SNPs were genotyped using a PCR-ligation detection reaction (LDR) method as previously described (3).

### Gene Expression Analysis

Gene Expression Profiling Interactive Analysis (GEPIA) was used to analyze the expression levels of IL15, IL17RA, AGER, MIF, and TNFRSF1A in 483 lung adenocarcinoma (LUAD) and 387 normal tissues from The Cancer Genome Atlas (TCGA) and the Genotype-Tissue Expression (GTEx) portal (31). The relationship between the expression levels of IL15, IL17RA, AGER, MIF, and TNFRSF1A and EGFR mutation status was analyzed using UCSC Xena (32). The progression-free survival (PFS) and overall survival (OS) were also analysis using UCSC Xena.

### Statistical Analyses

Weighted genetic risk score (GRS) was constructed to evaluate the cumulative effect of SNPs on the prognosis of NSCLC patients. Only SNPs with *P* value less than 0.05 entered the GRS with a coding value of 0 for non-risk allele homozygotes, 1 for heterozygotes, and 2 for risk-allele homozygotes. For each subject, we generated a GRS according to the formula: $GRS=\Sigma_{i=1}^{n}\;SNP_{i}\times \beta_{i}$ . β_i_ was the HR of the SNPi, whereas SNPi represented its code value. All patients were divided into low (1st quintile), intermediate (2nd–3th quintile), and high (4th quintile) GRS groups. PFS and OS rates were calculated using the Kaplan–Meier method. The differences in PFS and OS rates across different genotypes were compared using the log-rank test. Cox proportional hazard models were used to calculate hazard ratios (HRs) and 95% confidence intervals (CIs) for the evaluation of the association between SNPs and survival. A *P* value < 0.05 was considered statistically significant.

## Results

### Clinicopathologic Characteristics

Clinicopathologic characteristics of NSCLC patients were presented in **Table 1**. In brief, most of patients were female (66.7%). Mean age at diagnosis was 56.7 years. The median survival time was 22.0 months. A total of 182 (57.2%) patients died during follow-up period. TNM stage was significantly associated with both worse PFS (*P* = 0.020) and OS (*P* = 0.005). In addition, ECGO was week but significantly associated with worse OS (*P* = 0.047). There was no significant difference in PFS regarding to sex, age, ECGO, and smoking status (*P* > 0.05). There was also no significant difference in mortality rate regarding to sex, age, and smoking status (*P* > 0.05).

**Table 1:** Characteristics of the study populations at the time of analysis.

Variables	n (%)
Age (years)	
median (range)	57 (28-78)
Sex	
male	106 (33.3)
female	212 (66.7)
Smoking status	
nonsmoker	251 (78.9)
smoker	67 (21.1)
ECGO	
0-1	288 (90.6)
2	30 (9.4)
TNM stage	
IIIB	102 (32.1)
IV	216 (67.9)

ECOG, European Cooperative Eastern Group Classification.

### Associations Between SNPs and Survival Outcomes

**Figure 1:**
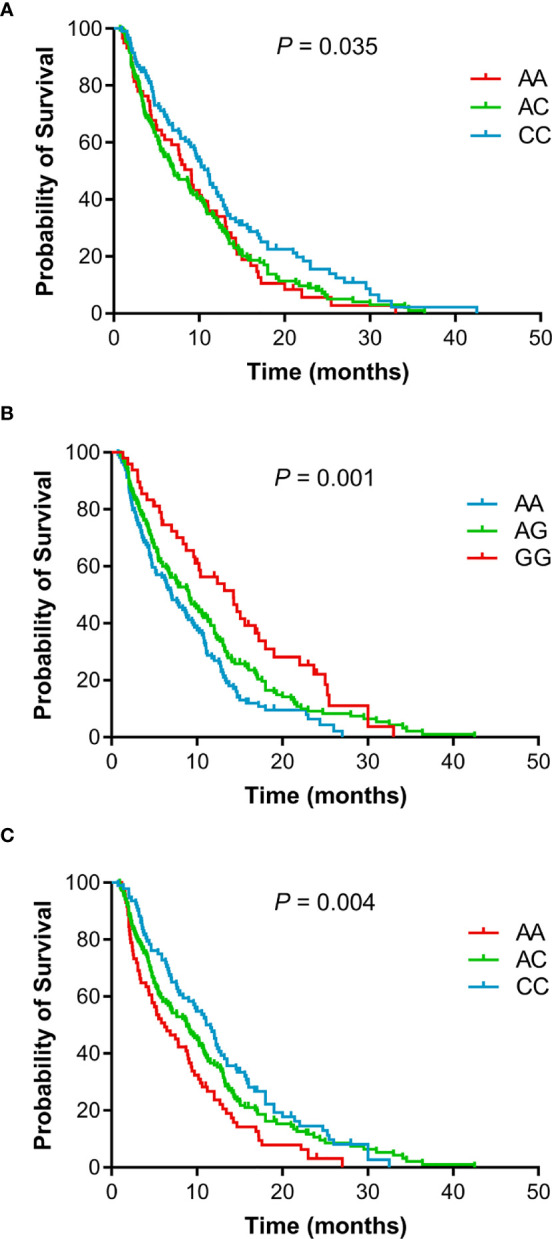
Kaplan-Meier curves of PFS for advanced NSCLC patients treated with EGFR TKIs according to genotypes. **(A)**, rs10519613. **(B)**, rs4819554. **(C)**, rs4149570.

**Figure 2:**
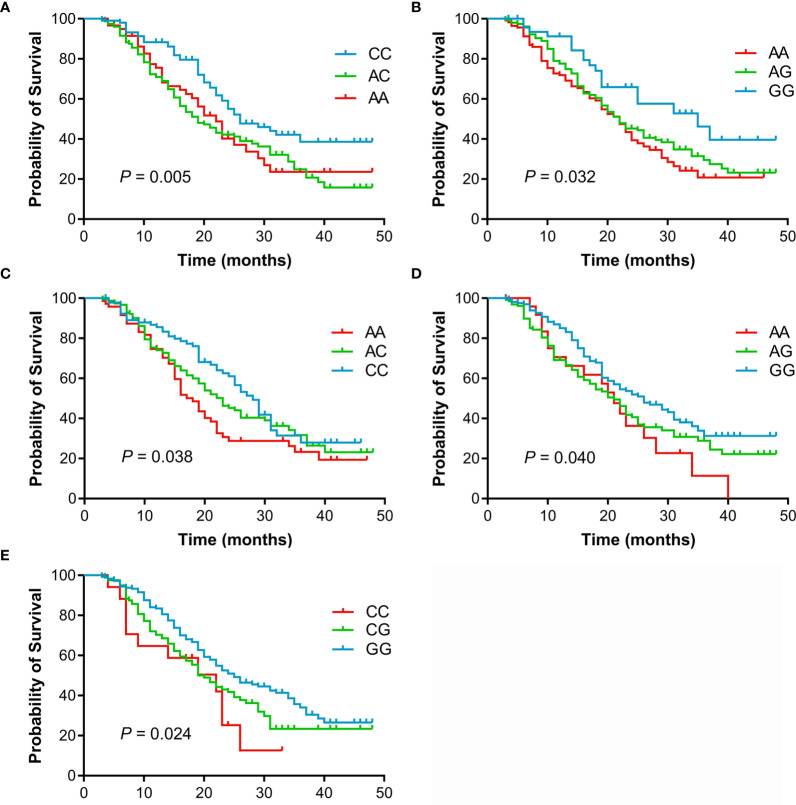
Kaplan-Meier curves of OS for advanced NSCLC patients treated with EGFR TKIs according to genotypes. **(A)**, rs10519613. **(B)**, rs4819554. **(C)**, rs4149570. **(D)**, rs2070600. **(E)**, rs755622.

The individual association of each SNP with the prognosis of NSCLC patients was shown in **Table 2**. Among 47 SNPs, 3 SNPs (rs10519613, rs4819554, and rs4149570) were significantly associated with worse PFS of NSCLC patients (**Figure 1**). Multivariate Cox’s proportional hazards regression analysis showed that rs10519613 (adjusted HR = 1.209, 95% CI 1.028-1.422, *P* = 0.022), rs4819554 (adjusted HR = 1.373, 95% CI 1.151-1.637, *P*  <  0.001), and rs4149570 (adjusted HR = 1.342, 95% CI 1.134-1.587, *P* = 0.001) were independent prognostic factors for worse PFS of NSCLC patients (**Table 2**). Furthermore, 5 SNPs (rs10519613, rs4819554, rs2070600, rs755622, and rs4149570) were significantly with worse OS of NSCLC patients (**Figure 2**). Multivariate Cox’s proportional hazards regression analysis showed that rs10519613 (adjusted HR = 1.282, 95% CI 1.047-1.569, *P* = 0.016), rs4819554 (adjusted HR = 1.319, 95% CI 1.060-1.641, *P* = 0.013), rs2070600 (adjusted HR = 1.333, 95% CI 1.068-1.664, *P* = 0.011), rs755622 (adjusted HR = 1.345, 95% CI 1.057-1.713, *P* = 0.016), and rs4149570 (adjusted HR = 1.328, 95% CI 1.083-1.627, *P* = 0.006) were independent prognostic factors for worse OS of NSCLC patients.

**Table 2:** Associations of SNPs of inflammation genes with PFS and OS of advanced lung adenocarcinoma patients treated with EGFR-TKIs.

Genes	SNPs	PFS*		OS*	
		HR (95% CI)	*P* value	HR (95% CI)	*P* value
IL1B	rs16944	0.991 (0.846-1.161)	0.914	0.953 (0.784-1.160)	0.632
IL2	rs2069762	0.971 (0.806-1.168)	0.753	1.022 (0.808-1.294)	0.853
IL2RA	rs2104286	1.155 (0.920-1.451)	0.214	1.011 (0.757-1.350)	0.941
IL4	rs2243250	1.013 (0.840-1.220)	0.896	1.070 (0.845-1.356)	0.575
IL6	rs1800796	1.031 (0.846-1.257)	0.761	1.006 (0.782-1.294)	0.961
IL10	rs1800896	1.076 (0.748-1.547)	0.695	1.311 (0.815-2.109	0.264
IL15	rs10519613	1.209 (1.028-1.422)	**0.022**	1.282 (1.047-1.569)	**0.016**
IL17A	rs2275913	0.940 (0.800-1.106)	0.457	0.913 (0.744-1.124)	0.386
IL17F	rs763780	1.094 (0.893-1.340)	0.386	1.102 (0.856-1.418)	0.451
IL17RA	rs4819554	1.373 (1.151-1.637)	**< 0.001**	1.319 (1.060-1.641)	**0.013**
IL21R	rs3093390	0.976 (0.740-1.289)	0.866	0.961 (0.686-1.348)	0.819
IL23R	rs10889677	0.926 (0.771-1.112)	0.409	1.141 (0.905-1.439)	0.264
IL32	rs28372698	0.894 (0.748-1.069)	0.218	0.866 (0.693-1.081)	0.203
PTGS2	rs5275	0.871 (0.707-1.073)	0.194	0.911 (0.701-1.184)	0.485
CXCL8	rs4073	0.944 (0.793-1.124)	0.515	0.898 (0.720-1.120)	0.341
CCR9	rs7613548	1.172 (0.991-1.386)	0.069	1.049 (0.851-1.292)	0.655
TNF	rs1800629	0.795 (0.611-1.035)	0.089	0.878 (0.628-1.227)	0.446
AURKA	rs2273535	0.919 (0.768-1.100)	0.356	0.862 (0.687-1.081)	0.198
PIN1	rs2287839	0.893 (0.607-1.314)	0.566	0.770 (0.484-1.226)	0.271
ITGA2	rs1126643	1.031 (0.861-1.236)	0.737	1.065 (0.853-1.331)	0.576
IL1R1	rs2234650	0.949 (0.796-1.131)	0.556	0.912 (0.734-1.132)	0.404
IL1R2	rs2071008	0.950 (0.792-1.140)	0.581	1.010 (0.800-1.274)	0.936
TGFB1	rs1800470	0.972 (0.815-1.158)	0.747)	0.961 (0.774-1.195)	0.723
AGER	rs2070600	1.108 (0.924-1.328)	0.268	1.333 (1.068-1.664)	**0.011**
MIF	rs755622	1.208 (0.996-1.465)	0.055	1.345 (1.057-1.713)	**0.016**
ICAM1	rs5498	0.901 (0.741-1.097)	0.299	1.056 (0.828-1.346)	0.663
CCR6	rs3093024	1.099 (0.915-1.320)	0.313	0.994 (0.790-1.250)	0.959
TLR4	rs1057317	0.943 (0.795-1.117)	0.495	1.132 (0.916-1.400)	0.252
CCR5	rs1799987	1.016 (0.843-1.223)	0.869	1.112 (0.886-1.394)	0.360
RNASEL	rs486907	0.894 (0.741-1.080)	0.245	0.871 (0.687-1.117)	0.284
IL10RA	rs9610	0.892 (0.748-1.063)	0.202	0.936 (0.755-1.160)	0.545
TNFRSF1B	rs1061624	0.947 (0.808-1.109)	0.497	0.906 (0.741-1.107)	0.333
CAPN10	rs3792267	1.230 (0.968-1.564)	0.091	1.241 (0.912-1.689)	0.170
CRP	rs1205	0.917 (0.777-1.082)	0.303	0.935 (0.760-1.150)	0.524
NFKB1	rs28362491	1.075 (0.914-1.265)	0.382	1.037 (0.846-1.270)	0.728
IL21	rs907715	1.026 (0.876-1.201)	0.752	1.064 (0.876-1.293)	0.531
CSF1R	rs10079250	1.100 (0.920-1.314)	0.295	1.172 (0.94-1.470)	0.171
CCL2	rs1024611	0.975 (0.835-1.138)	0.745	0.918 (0.754-1.117)	0.391
TREM1	rs2234246	1.120 (0.905-1.387)	0.299	0.970 (0.755-1.246)	0.811
PTX3	rs2305619	1.014 (0.862-1.193)	0.865	1.124 (0.915-1.380)	0.267
SOCS1	rs243327	1.144 (0.961-1.362)	0.131	1.215 (0.978-1.509)	0.079
IFNG	rs2430561	0.912 (0.763-1.090)	0.313	0.848 (0.684-1.052)	0.134
CXCR3	rs2280964	1.024 (0.872-1.202)	0.772	0.947 (0.777-1.155)	0.591
TNFRSF1A	rs4149570	1.342 (1.134-1.587)	**0.001**	1.328 (1.083-1.627)	**0.006**
PTGS1	rs3842787	1.048 (0.810-1.357)	0.720	0.918 (0.682-1.236)	0.573
PTGIR	rs1126510	0.932 (0.738-1.176)	0.552	1.074 (0.813-1.419)	0.614
ITGB3	rs2317385	1.100 (0.932-1.297)	0.260	1.059 (0.861-1.302)	0.588

CI, confidence interval; HR, hazard ratio; PFS, progression-free survival; OS, overall survival.

Statistically significant values are in bold.

*Adjusted by sex, age, smoking status, ECGO, and TNM stage.

### Associations Between GRS and Survival Outcomes

**Figure 3:**
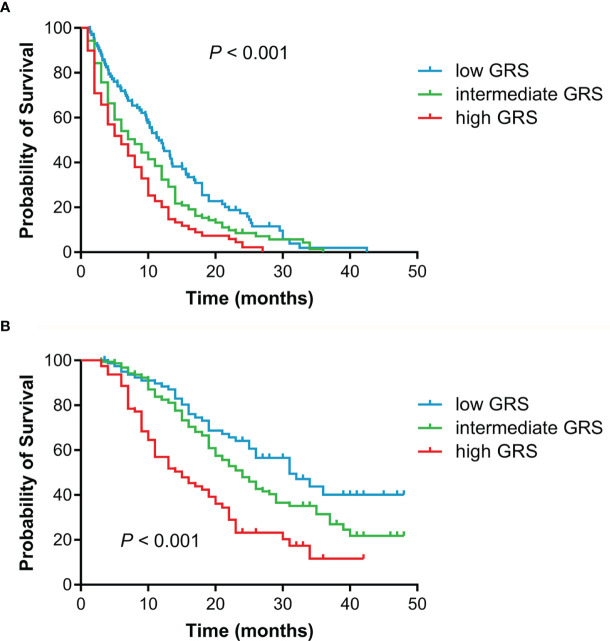
Kaplan-Meier survival curves for advanced NSCLC patients treated with EGFR TKIs according to GRSs. **(A)**, PFS. **(B)**, OS.

Survival analysis according to GRS was shown in **Table 3**. Patients with high GRS (based on rs10519613, rs4819554, and rs4149570) had significantly shorter PFS (6.5 months) than those with intermediate (7.9 months) and low GRSs (11.6 months, *P* < 0.001) (**Figure 3**). Multivariate Cox’s proportional hazards regression analysis showed that intermediate (adjusted HR = 1.432, 95%CI 1.082-1.897, *P* = 0.012) and high GRSs (adjusted HR = 2.008, 95%CI 1.453-2.774, *P* < 0.001) were significantly associated with worse PFS of NSCLC patients. We further also evaluated the association of GRS (based on rs10519613, rs4819554, rs2070600, rs755622, and rs4149570) with OS. Patients with high GRS had significantly shorter OS (15.0 months) than those with intermediate (24.0 months) and low GRSs (31.0 months, *P* < 0.001). Multivariate Cox’s proportional hazards regression analysis showed that high GRSs (adjusted HR = 2.816, 95%CI 1.845-4.300, *P* < 0.001) were significantly associated with worse OS of NSCLC patients.

**Table 3:** Associations of GRSs with PFS and OS of advanced lung adenocarcinoma patients treated with EGFR-TKIs.

GRS	PFS*			OS*		
	Events/n	HR (95% CI)	*P* value	Events/n	HR (95% CI)	*P* value
low	83/97	1		36/80	1	
intermediate	127/142	1.432 (1.082-1.897)	0.012	87/159	1.416 (0.953-2.105)	0.085
high	76/79	2.008 (1.453-2.774)	< 0.001	59/79	2.816 (1.845-4.300)	< 0.001

CI, confidence interval; GRS, genetic risk score; HR, hazard ratio; PFS, progression-free survival; OS, overall survival.

*Adjusted by sex, age, smoking status, ECGO, and TNM stages.

### Sex-Stratified Analysis

Previous studies have shown that female patients have higher frequency of EGFR mutation (33, 34), which suggested whether sex may influence the effect of SNPs on clinical outcome of NSCLC patients. We further conducted a sex-stratified analysis of the aforementioned statistically significant SNPs that were associated with the clinical outcome of NSCLC patients. Among female NSCLC patients, only high GRS was significantly associated with both PFS (adjusted HR = 1.629, 95%CI 1.105-2.400, *P* = 0.014) and OS (adjusted HR = 1.967, 95%CI 1.176-3.289, *P* = 0.010), and rs10519613 was weakly but significantly associated with worse OS (adjusted HR = 1.312, 95%CI 1.021-1.687, *P* = 0.034) (**Table 4**). Among male NSCLC patients, intermediate and high GRSs, rs4819554, and rs4149570 were significantly associated with both PFS and OS (*P* < 0.05). Furthermore, rs10519613 and rs755622 were significantly associated with worse PFS (adjusted HR = 1.392, 95%CI 1.034-1.874, *P* = 0.029) and OS (adjusted HR = 1.699, 95%CI 1.172-2.463, *P* = 0.005), respectively.

**Table 4:** Associations of SNPs and GRS with OS and PFS of advanced lung adenocarcinoma patients treated with EGFR-TKI stratified by sex.

Sex	SNPs/GRS	PFS*		OS*	
		HR (95% CI)	*P* value	HR (95% CI)	*P* value
Male	rs10519613	1.392 (1.034-1.874)	0.029	1.263 (0.897-1.779)	0.181
	rs4819554	1.566 (1.123-2.185)	0.008	1.677 (1.113-2.527)	0.013
	rs4149570	1.747 (1.267-2.410)	0.001	1.613 (1.112-2.340)	0.012
	rs2070600	–		1.399 (0.938-2.087)	0.099
	rs755622	–		1.699 (1.172-2.463)	0.005
	low GRS	1		1	
	Intermediate GRS	2.016 (1.167-3.482)	0.012	2.984 (1.445-6.159)	0.003
	high GRS	3.042 (1.653-5.599)	< 0.001	4.466 (2.115-9.430)	< 0.001
Female	rs10519613	1.152 (0.948-1.401)	0.155	1.312 (1.021-1.687)	0.034
	rs4819554	1.238 (1.000-1.533)	0.050	1.177 (0.902-1.535)	0.230
	rs4149570	1.172 (0.959-1.433)	0.122	1.123 (0.876-1.439)	0.361
	rs2070600	–		1.289 (0.991-1.676)	0.059
	rs755622	–		1.194 (0.878-1.624)	0.258
	low GRS	1		1	
	Intermediate GRS	1.288 (0.918-1.808)	0.143	0.834 (0.951-1.525)	0.834
	high GRS	1.629 (1.105-2.400)	0.014	1.967 (1.176-3.289)	0.010

CI, confidence interval; GRS, genetic risk score; HR, hazard ratio; PFS, progression-free survival; OS, overall survival.

*Adjusted by age, smoking status, ECGO and stages.

### Associations Between the Expression of IL15, IL17RA, AGER, MIF, and TNFRSF1A and Survival Outcomes

**Figure 4:**
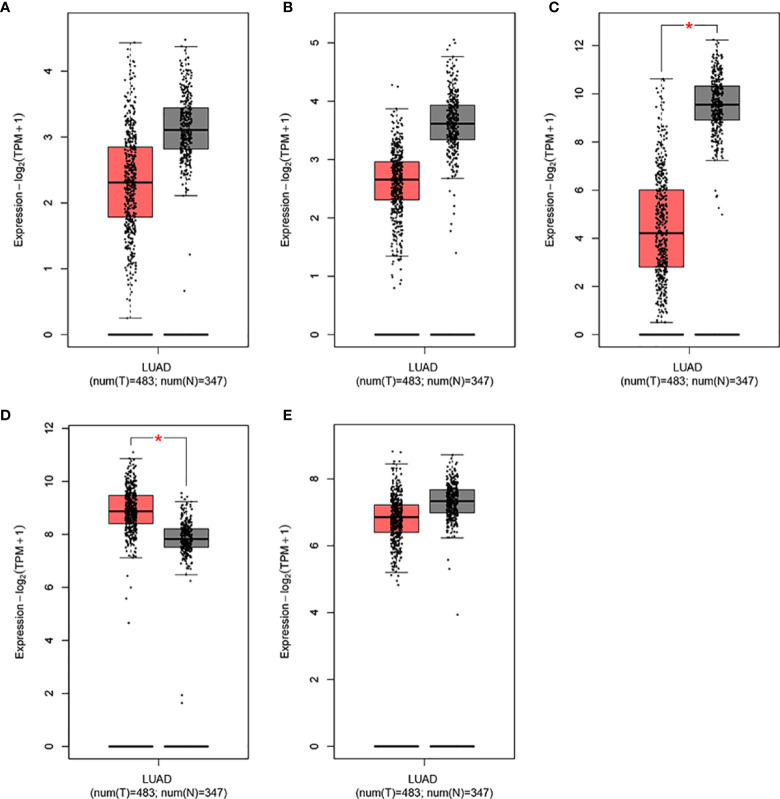
The expression levels of IL15, IL17RA, AGER, MIF, and TNFRSF1A in LUAD in GEPIA. **(A)**, IL15. **(B)**, IL17RA. **(C)**, AGER. **(D)**, MIF. **(E)**, TNFRSF1A. *|Log2FC| > 1.00 and P < 0.010.

**Figure 5:**
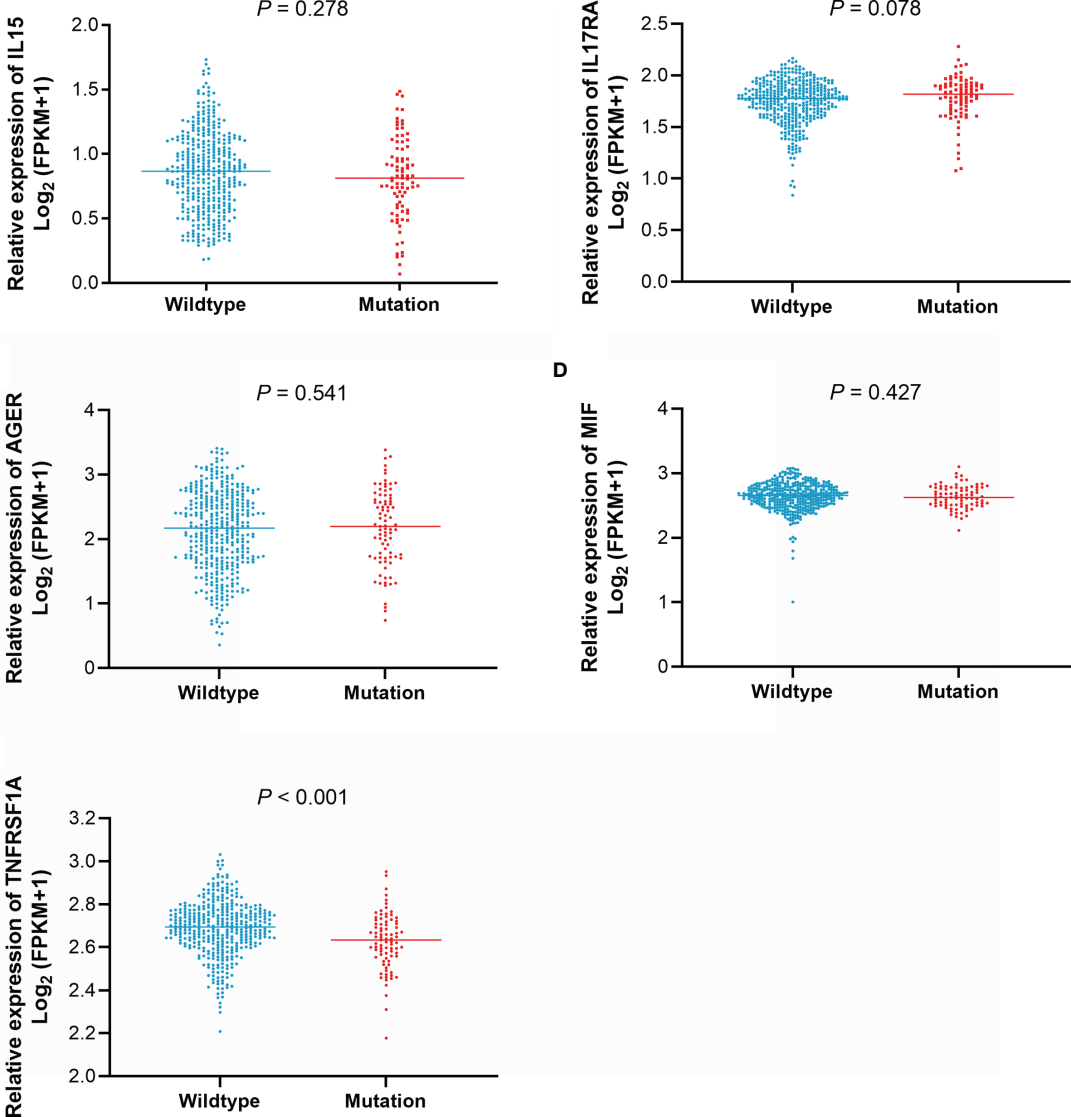
The expression levels of IL15, IL17RA, AGER, MIF, and TNFRSF1A according to EGFR mutation status. **(A)**, IL15. **(B)**, IL17RA. **(C)**, AGER. **(D)**, MIF. **(E)**, TNFRSF1A.

To determine differences in the expression levels of IL15, IL17RA, AGER, MIF, and TNFRSF1A in LUAD and normal tissues, their expression levels were analyzed using the GEPIA (31). As shown in **Figure 4**, IL15, IL17RA, AGER, and TNFRSF1A were significantly downregulated, whereas MIF was significantly upregulated in LUAD (n = 483) than those in normal tissues (n = 347). Furthermore, TNFRSF1A was significantly downregulated in EGFR-mutant LUAD (n = 85) than that in EGFR wildtype LUAD (n = 394) (*P* < 0.001). IL17RA was weakly upregulated in EGFR-mutant LUAD than that in EGFR wildtype LUAD, but not reached a significant level (*P* = 0.078, **Figure 5**). There was no difference between the expression levels of IL15, AGER, and MIF and EGFR mutation status (*P* > 0.05).

**Figure 6:**
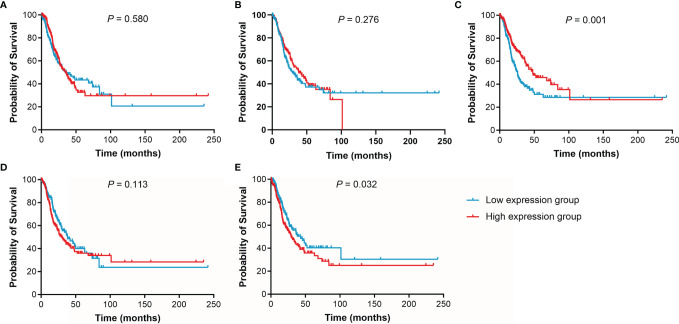
Kaplan-Meier curves of PFS for LUAD patients. **(A)**, IL15. **(B)**, IL17RA. **(C)**, AGER. **(D)**, MIF. **(E)**, TNFRSF1A.

**Figure 7:**
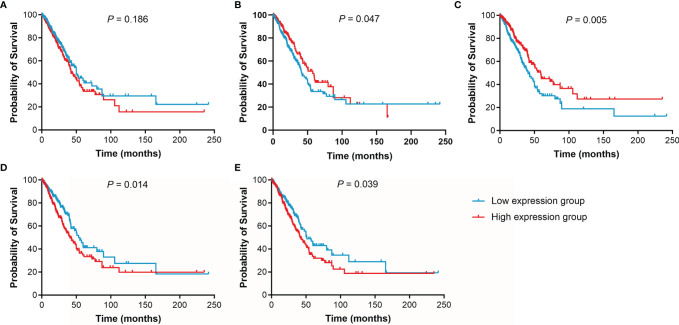
Kaplan-Meier curves of OS for LUAD patients. **(A)**, IL15. **(B)**, IL17RA. **(C)**, AGER. **(D)**, MIF. **(E)**, TNFRSF1A.

**Figure 8:**
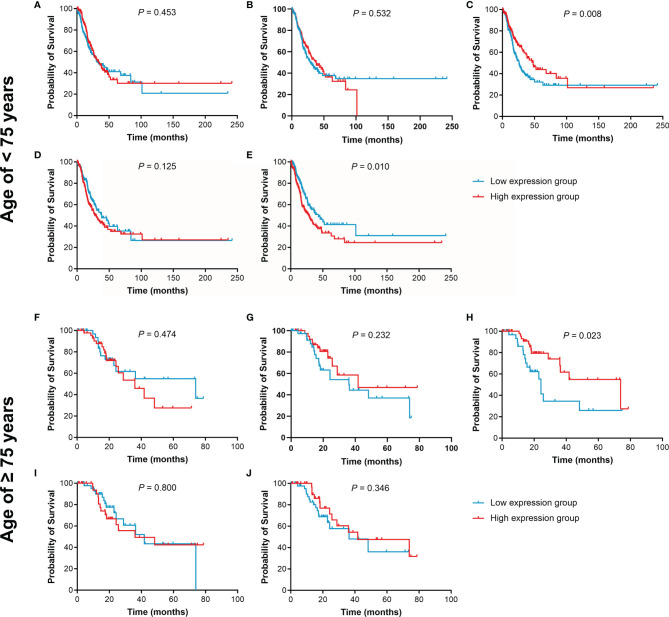
Kaplan-Meier curves of PFS for LUAD patients stratified by age. **(A, E)**, the PFS curves of LUAD patients younger than 75 years were assessed according to IL15, IL17RA, AGER, MIF, and TNFRSF1A expression, respectively. **(F–J)**, the PFS curves of LUAD patients aged 75 years or older were assessed according to IL15, IL17RA, AGER, MIF, and TNFRSF1A expression, respectively.

**Figure 9:**
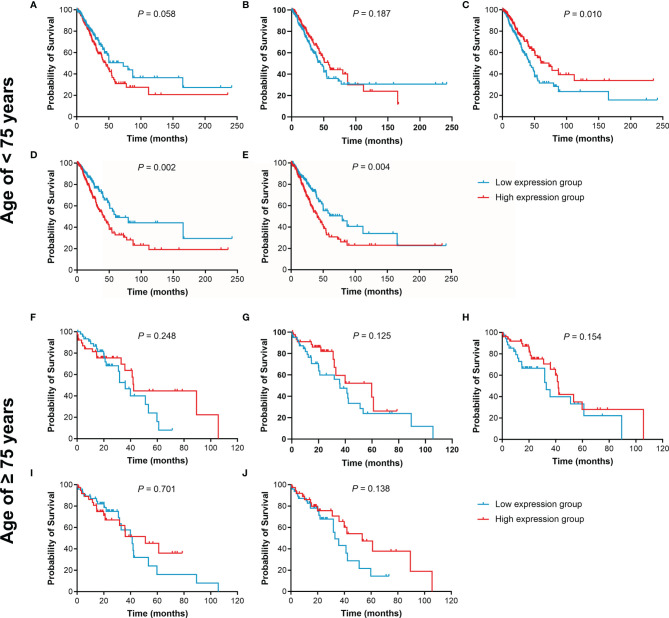
Kaplan-Meier curves of OS for LUAD patients stratified by age. **(A–E)**, the OS curves of LUAD patients younger than 75 years were assessed according to IL15, IL17RA, AGER, MIF, and TNFRSF1A expression, respectively. **(F–J)**, the OS curves of LUAD patients aged 75 years or older were assessed according to IL15, IL17RA, AGER, MIF, and TNFRSF1A expression, respectively.

The effect of IL15, IL17RA, AGER, MIF, and TNFRSF1A expression on survival in LUAD was also analyzed using UCSC Xena. Both low AGER expression and high TNFRSF1A expression were significantly associated with worse PFS in LUAD (*P* < 0.05, **Figure 6**). No difference in PFS was observed between low and high IL15, IL17RA, and MIF expression groups (*P* > 0.05). In addition, low IL17RA and AGER expression, high MIF and TNFRSF1A expression were significantly associated with worse OS in LUAD (*P* < 0.05, **Figure 7**). No difference in OS was observed between low and high IL15 expression groups (*P* = 0.186). Given that the degree of systemic inflammation in elderly patients may influence the response to anticancer treatment (35, 36), we further evaluated the effects of IL15, IL17RA, AGER, MIF, and TNFRSF1A expression on clinical outcome of LUAD patients stratified by age. Low AGER expression was significantly associated with shorter PFS in both patients younger than 75 years and those aged 75 years or older (*P* < 0.05), whereas high TNFRSF1A expression was only significantly associated with shorter PFS in patients younger than 75 years (*P* = 0.010, **Figure 8**). In addition, low AGER expression, high MIF and TNFRSF1A expression were significantly associated with shorter OS only in patients younger than 75 years (*P* < 0.05, **Figure 9**).

## Discussion

The EGFR mutation rate in NSCLC is relatively high (~50.2%) and thus the EGFR-TKIs are the most commonly used targeted drugs for the treatment of NSCLC (6, 7). Although the PFS and OS of osimertinib are significantly longer than those of the first- and second-generation EGFR-TKIs, the problem of acquired osimertinib resistance still exists in long-term use (8, 12, 37). How to improve or maintain the sensitivity of EGFR-TKIs, delays the onset of resistance, and overcomes acquired resistance to EGFR TKIs in NSCLC patients is still an area with unmet high clinical needs. In the present study, we evaluated the association of SNPs in inflammation genes with the prognosis of advanced NSCLC patients treated with EGFR-TKIs. SNPs in inflammation genes were associated with worse PFS and OS of NSCLC patients. Therefore, SNPs in inflammation genes may be independent prognostic factors for poor prognosis of NSCLC patients treated with EGFR-TKIs.

Inflammation play a critical role in the development and progression of cancer (15, 16). The field of cancer-related inflammation has expanded tremendously over the past decade, and the underlying cellular and molecular mechanisms have been being unraveled one after another. Inflammatory markers may be used as clinically prognostic markers for predicting outcome and monitoring the cancer progression, whereas anti-inflammatory therapy may be a potential regimen for cancer treatment (27, 28, 38, 39). It is estimated that approximately 25% of cancer cases are related to infectious diseases and chronic inflammation (40). The reduction of inflammatory molecule production has been shown to enhance the sensitivity of NSCLC with EGFR mutation to EGFR-TKIs (41). In support of the hypothesis that inflammation is associated with cancer development and progression and also influence the response to treatment, we found that 3 SNPs (rs10519613, rs4819554, and rs4149570) and 5 SNPs (rs10519613, rs4819554, rs2070600, rs755622, and rs4149570) were associated with worse PFS and OS of advanced NSCLC patients treated with EGFR-TKIs, respectively. The effect was increased in NSCLC patients with intermediate and high GRSs, indicating that these patients may have higher levels of systemic inflammation. These findings imply that among patients with intermediate and high GRSs, there is less clinical benefit from EGFR-TKI therapy, as might be expected that this effect is weakened by inflammation. Patients with intermediate and high GRSs seem to be more likely to develop resistance to EGFR-TKIs and may have a high risk of developing EGFR-TKI-induced adverse events such as pneumonitis. SNPs in inflammation genes could discriminate the clinical outcome of NSCLC patients and might affect follow-up and therapeutic strategies after treated with EGFR-TKIs.

A study by Umekawa et al. (20) revealed that EGFR-TKIs may inhibit cancer-related inflammatory response and tumor growth, and improve the prognosis and quality of life of patients. A recent study by Jia et al. (42) found that EGFR-TKIs can modify the tumor microenvironment (TME) during which the levels of cytotoxic CD8^+^ T cells and dendritic cells (DCs) were rapidly elevated, Foxp3^+^ regulatory T cells (Tregs) were eliminated, and M2-like polarization of macrophages was suppressed at an early stage, which were only temporary and disappeared as treatment continued. However, long-term EGFR-TKI treatment leads to the transformation of tumor immunosurveillance into tumor-promoting inflammation through the induction of Tregs, inhibition of DCs, and the polarization of macrophages toward the M2 phenotype (42, 43). MIF is a pro-inflammatory cytokine that functions as a critical molecule in the innate immune system and involves in carcinogenesis, cancer cell proliferation, invasion, metastasis, angiogenesis, and drug resistance, and dampens the anti-tumor immune response (44–49). Our analysis also revealed that high MIF expression was associated with worse prognosis in LUAD cohort. Furthermore, rs755622 were significantly with worse prognosis of NSCLC patients treated with EGFR-TKI. rs755622 in the promoter of MIF affects transcription activity that subsequently results in an increased expression of MIF (50). Therefore, elevated MIF not only suppresses immune surveillance and anti-tumor immune responses and promotes tumor growth, but also favors cancer cell survival against anticancer drugs (46, 47, 49). Inhibition of MIF not only suppresses cancer cell proliferation, but also enhance the sensitivity of cancer cells to anticancer drugs, including EGFR-TKIs, and overcomes anticancer drug resistance (45–48).

Tumor necrosis factor (TNF) is a key regulator of innate immunity and plays a paradoxical and dual role in human cancers, promoting cancer survival or cell death depending on the cellular context (51). The pleiotropic activities of TNF are mediated through two distinct cell surface receptors, TNFRSF1A and TNFRSF1B. Some studies have shown that TNF signaling pathway contributes to the development of drug resistance in NSCLC, breast cancer, and clear cell renal cell carcinoma (52–54). TNF inhibition *via* TNFRSF1A silencing, etanercept, or thalidomide increases sensitivity of lung cancer cells to EGFR-TKIs, whereas TNF overexpression attenuates apoptosis induction by EGFR-TKIs (54). Although TNFRSF1A was significantly downregulated in TCGA LUAD cohort, high TNFRSF1A expression predicted a worse prognosis. rs4149570 in the promoter of TNFRSF1A affects its expression level (55). Lee et al. (52) reported that rs4149570 was associated with worse prognosis in patients with early-stage NSCLC. In this study, rs4149570 was also associated with worse prognosis in advanced NSCLC patients treated with EGFR-TKI. These pieces of evidence indicate that TNF signaling pathway is extensively implicated in the progression and treatment response in NSCLC.

IL15 is a pleiotropic cytokine best known for its ability to induce T and natural killer (NK) cell activation and proliferation. Combination of IL15 or its superagonists with immune checkpoint inhibitors showed obvious antitumor effects (56). Two studies showed that rs10519613 in IL15 was associated with the risk of minimal residual disease (MRD) in pediatric acute lymphoblastic leukemia (57, 58). Zhang et al. (59) observed that rs10519613 was associated with poor prognosis of HBV-related hepatocellular carcinoma after liver transplantation. In this study, we found that rs10519613 was associated with worse PFS and OS in advanced NSCLC patients treated with EGFR-TKI. The function of rs10519613 is unknown and thus further studies are required to elucidate its function.

IL17 is a T cell-derived proinflammatory cytokine that is essential for a variety of processes such as innate immune defenses, tissue repair, the pathogenesis of autoimmune and inflammatory diseases, and cancer progression (60). IL17RA is one of five IL17 receptors (IL-17RA to IL-17RE) and is widely expressed in a variety of tissues. Two studies reported that the protein level of IL-17RA were significantly increased in NSCLC tissues (61, 62), whereas Liu et al. (63) found no difference in IL-17RA protein expression between NSCLC and normal lung tissues. However, IL17RA was significantly downregulated in TCGA LUAD cohort. This inconsistency may be partly due to tumor heterogeneity and different detection techniques. rs4819554 in the promoter of IL-17RA affects its expression levels and is associated with susceptibilities of some diseases such as autoimmune diseases and cancer (62, 64–66). Furthermore, rs4819554 was significantly associated with the response to anti-TNF drugs in psoriasis patients (67). Given that high expression levels of IL17RA in EGFR-mutated LUAD, NSCLC patients treated with EGFR-TKIs with risk allele seem to have higher expression levels of IL17RA, indicating elevated levels of proinflammatory cytokines in NSCLC tissues (61). This may attribute to the progression of lung cancer and the development of acquired resistances during therapy.

AGER is a multiligand cell surface receptor that involves in initiating of pro-inflammatory intracellular signaling pathways (68). AGER signaling pathway plays an important role in diverse physiological and pathophysiological processes and diseases such as autoimmune diseases and cancer. Previous studies have shown that AGER is downregulated in lung cancer and exhibits tumor suppression function (69–71). Furthermore, knockdown of AGER decreases both the quantity and suppressive activity of tumor-induced myeloid-derived suppressor cells (MDSCs) (72). However, the infiltration of MDSCs is consistently increased throughout the periods of EGFR-TKI treatment, which can inhibit antitumor immune responses, facilitate cancer progression, and lead to EGFR-TKI resistance (42, 73). rs2070600 may promote glycosylation of AGER and affect its splicing (74, 75). Many studies have demonstrated that rs2070600 is associated with many diseases such as autoimmune diseases and lung cancer (68, 71, 76). Yamaguchi et al. (76) found that rs2070600 was related to systemic inflammation and poor prognosis of lung cancer. Therefore, high level of systemic inflammation not only means that NSCLC patients may have more adverse risk factors, but also that NSCLC patients may have worse clinical outcome when treated with EGFR-TKIs.

The inherited genetic information is increasingly being used to predict response to therapy and aid in treatment decisions in clinical practice. However, there are very limited data available that inherited genetic variations can predict clinical outcome after treated with EGFR-TKIs. Given extensive intra-tumor and inter-tumor heterogeneity, it is attractive to use inherited genetic variation to predict response, which could allow for more effective tailored treatment for NSCLC patients. Indeed, our finding that SNPs in inflammation genes may influence the benefits of EGFR-TKIs treatment in NSCLC patients is interesting and deserves further investigation. These polymorphisms may affect the baseline level of systemic inflammation, which could potentially impair the efficacy of EGFR-TKIs and enhance the development of resistance to EGFR-TKIs. In addition, there are some limitations in our study that could not be ignored and suggest caution in the interpretation of the findings. First, the sample size was relatively small, which does not provide us with adequate statistical power to rule out the natural variation existing between individuals. Second, NSCLC patients in our study were all unresectable advanced patients. Therefore, the value of these SNPs in resectable NSCLC patients is required to be evaluated. Third, our results preliminarily revealed that the expression levels of inflammatory genes mainly affected the prognosis of patients younger than 75 years not elderly patients aged 75 years or older. Whether SNPs of inflammation genes play different roles in the elderly patients remains to be further evaluated due to only 6 patients aged 75 years or older in the present study. Fourth, the lack of circulating inflammatory biomarkers (such as CRP and cytokines) data makes it impossible to conduct a comprehensive analysis in conjunction with patients’ baseline inflammatory status. Despite these limitations, this study is one of the very few studies to date exploring the impact of inherited genetic variations on the prognosis of advanced NSCLC patients treated with EGFR-TKIs.

## Conclusion

We identified 3 SNPs (rs10519613, rs4819554, and rs4149570) and 5 SNPs (rs10519613, rs4819554, rs2070600, rs755622, and rs4149570) in inflammation genes that were prognostic indicators for PFS and OS of advanced NSCLC treated with EGFR-TKIs, respectively. Although further studies are required to confirm the association and elucidate its underlying molecular mechanism, our findings provided evidence that SNPs in inflammation genes may have the potential to serve as prognostic biomarkers for NSCLC in the clinical setting. Further large-scale studies are necessary to validate the applicability of these SNPs as predictive biomarkers in NSCLC patients.

## Data Availability Statement

The original contributions presented in the study are included in the article/**Supplementary Material**. Further inquiries can be directed to the corresponding authors.

## Ethics Statement

The studies involving human participants were reviewed and approved by the Ethics Committees of Taizhou Central Hospital. The patients/participants provided their written informed consent to participate in this study.

## Author Contributions

XZ, HS, and CH designed the research. TY, ML, HL and HY performed the research. XZ, HSL, ZQ and CH provided the clinical samples. XZ, HS, and CH analyzed and interpreted the data, and wrote the paper. HS revised the final version of the manuscript. All authors approved the final version of the manuscript.

## Funding

This study was supported by the Taizhou Science and Technology Agency, China (Grant 162yw02-4).

## Conflict of Interest

The authors declare that the research was conducted in the absence of any commercial or financial relationships that could be construed as a potential conflict of interest.

## Publisher’s Note

All claims expressed in this article are solely those of the authors and do not necessarily represent those of their affiliated organizations, or those of the publisher, the editors and the reviewers. Any product that may be evaluated in this article, or claim that may be made by its manufacturer, is not guaranteed or endorsed by the publisher.
