# Supplementary material for: Association of Polymorphisms in Inflammation Genes With the Prognosis of Advanced Non-Small Cell Lung Cancer Patients Receiving Epidermal Growth Factor Receptor Tyrosine Kinase Inhibitors
Source: Front Oncol. 2022 Mar 18;12:836117. doi: 10.3389/fonc.2022.836117 (PMC8971721; doi:10.3389/fonc.2022.836117)
Supplement: Supplementary file 1 [file Table_1.docx]

| **TABLE S1** Candidate genes and SNPs included in the study |  |  |  |  |  |
| --- | --- | --- | --- | --- | --- |
| **Genes** | **Symbol** | **SNPs** | **Minor Allele** | **MAF** | **HWE** |
| interleukin 15 | IL15 | rs10519613 | A | 0.43 | 0.848 |
| interleukin 17 receptor A | IL17RA | rs4819554 | G | 0.40 | 0.652 |
| interleukin 21 receptor | IL21R | rs3093390 | T | 0.10 | 0.727 |
| interleukin 23 receptor | IL23R | rs10889677 | C | 0.27 | 0.394 |
| interleukin 1 beta | IL1B | rs16944 | A | 0.48 | 0.099 |
| prostaglandin-endoperoxide synthase 2 | PTGS2 | rs5275 | G | 0.20 | 0.315 |
| C-X-C motif chemokine ligand 8 | CXCL8 | rs4073 | A | 0.38 | 0.151 |
| interleukin 17A | IL17A | rs2275913 | G | 0.46 | 0.370 |
| interleukin 10 | IL10 | rs1800896 | G | 0.06 | 0.244 |
| interleukin 32 | IL32 | rs28372698 | T | 0.33 | 0.757 |
| C-C motif chemokine receptor 9 | CCR9 | rs7613548 | A | 0.37 | 0.800 |
| tumor necrosis factor | TNF | rs1800629 | A | 0.12 | 0.326 |
| aurora kinase A | AURKA | rs2273535 | T | 0.37 | 0.707 |
| peptidylprolyl cis/trans isomerase, NIMA-interacting 1 | PIN1 | rs2287839 | C | 0.05 | 0.345 |
| integrin subunit alpha 2 | ITGA2 | rs1126643 | A | 0.32 | 0.631 |
| interleukin 17F | IL17F | rs763780 | C | 0.19 | 0.745 |
| interleukin 6 | IL6 | rs1800796 | G | 0.26 | 0.789 |
| interleukin 1 receptor type 1 | IL1R1 | rs2234650 | T | 0.39 | 0.112 |
| interleukin 1 receptor type 2 | IL1R2 | rs2071008 | T | 0.26 | 0.682 |
| transforming growth factor beta 1 | TGFB1 | rs1800470 | C | 0.47 | 0.062 |
| advanced glycosylation end-product specific receptor | AGER | rs2070600 | A | 0.28 | 0.958 |
| macrophage migration inhibitory factor | MIF | rs755622 | C | 0.25 | 0.518 |
| interleukin 2 | IL2 | rs2069762 | G | 0.23 | 0.342 |
| interleukin 2 receptor subunit alpha | IL2RA | rs2104286 | G | 0.15 | 0.590 |
| interleukin 4 | IL4 | rs2243250 | C | 0.23 | 0.881 |
| intercellular adhesion molecule 1 | ICAM1 | rs5498 | G | 0.30 | 0.068 |
| C-C motif chemokine receptor 6 | CCR6 | rs3093024 | A | 0.37 | 0.278 |
| toll like receptor 4 | TLR4 | rs1057317 | A | 0.42 | 0.962 |
| C-C motif chemokine receptor 5 | CCR5 | rs1799987 | A | 0.43 | 0.082 |
| ribonuclease L | RNASEL | rs486907 | A | 0.25 | 0.484 |
| interleukin 10 receptor subunit alpha | IL10RA | rs9610 | G | 0.30 | 0.133 |
| TNF receptor superfamily member 1B | TNFRSF1B | rs1061624 | G | 0.50 | 0.737 |
| calpain 10 | CAPN10 | rs3792267 | A | 0.14 | 0.719 |
| C-reactive protein | CRP | rs1205 | G | 0.44 | 0.501 |
| nuclear factor kappa B subunit 1 | NFKB1 | rs28362491 | D | 0.47 | 0.465 |
| interleukin 21 | IL21 | rs907715 | A | 0.47 | 0.294 |
| colony stimulating factor 1 receptor | CSF1R | rs10079250 | G | 0.40 | 0.292 |
| C-C motif chemokine ligand 2 | CCL2 | rs1024611 | T | 0.43 | 0.085 |
| triggering receptor expressed on myeloid cells 1 | TREM1 | rs2234246 | A | 0.20 | 0.460 |
| pentraxin 3 | PTX3 | rs2305619 | A | 0.36 | 0.799 |
| suppressor of cytokine signaling 1 | SOCS1 | rs243327 | A | 0.28 | 0.921 |
| interferon gamma | IFNG | rs2430561 | T | 0.28 | 0.096 |
| C-X-C motif chemokine receptor 3 | CXCR3 | rs2280964 | T | 0.39 | 0.064 |
| TNF receptor superfamily member 1A | TNFRSF1A | rs4149570 | A | 0.47 | 0.633 |
| prostaglandin-endoperoxide synthase 1 | PTGS1 | rs3842787 | T | 0.12 | 0.353 |
| prostaglandin I2 receptor | PTGIR | rs1126510 | A | 0.14 | 0.865 |
| integrin subunit beta 3 | ITGB3 | rs2317385 | T | 0.46 | 0.067 |
| MAF, minor allele frequency; HWE, Hardy-Weinberg equilibrium | | | | | |
